# Treatment with Cobra Venom Factor Decreases Ischemic Tissue Damage in Mice

**DOI:** 10.3390/biomedicines12020309

**Published:** 2024-01-29

**Authors:** Sharon O. Azubuike-Osu, Amelie Kuhs, Philipp Götz, Anna Faro, Klaus T. Preissner, Christoph Arnholdt, Elisabeth Deindl

**Affiliations:** 1 Walter-Brendel-Centre of Experimental Medicine, University Hospital, Ludwig-Maximilians-Universität München, 81377 Munich, Germany or sharon.eboagwu@funai.edu.ng (S.O.A.-O.); amelie.kuhs@campus.lmu.de (A.K.); p.goetz@med.uni-muenchen.de (P.G.); anna.braumandl@web.de (A.F.); christoph.arnholdt@med.uni-muenchen.de (C.A.); 2 Biomedical Center, Institute of Cardiovascular Physiology and Pathophysiology, Ludwig-Maximilians-Universität München, 82152 Planegg-Martinsried, Germany; 3 Department of Physiology, Faculty of Basic Medical Sciences, College of Medicine, Alex Ekwueme Federal University Ndufu Alike, Abakaliki 482131, Ebonyi, Nigeria; 4 Department of Cardiology, Kerckhoff-Heart Research Institute, Faculty of Medicine, Justus Liebig University, 35392 Giessen, Germany; klaus.t.preissner@biochemie.med.uni-giessen.de

**Keywords:** ischemia, angiogenesis, cobra venom factor, complement system, leukocytes, macrophages, macrophage polarization, vascular occlusion, inflammation

## Abstract

Tissue ischemia, caused by the blockage of blood vessels, can result in substantial damage and impaired tissue performance. Information regarding the functional contribution of the complement system in the context of ischemia and angiogenesis is lacking. To investigate the influence of complement activation and depletion upon femoral artery ligation (FAL), Cobra venom factor (CVF) (that functionally resembles C3b, the activated form of complement component C3) was applied in mice in comparison to control mice. Seven days after induction of muscle ischemia through FAL, gastrocnemius muscles of mice were excised and subjected to (immuno-)histological analyses. H&E and apoptotic cell staining (TUNEL) staining revealed a significant reduction in ischemic tissue damage in CVF-treated mice compared to controls. The control mice, however, exhibited a significantly higher capillary-to-muscle fiber ratio and a higher number of proliferating endothelial cells (CD31^+^/CD45^−^/BrdU^+^). The total number of leukocytes (CD45^+^) substantially decreased in CVF-treated mice versus control mice. Moreover, the CVF-treated group displayed a shift towards the M2-like anti-inflammatory and regenerative macrophage phenotype (CD68^+^/MRC1^+^). In conclusion, our findings suggest that treatment with CVF leads to reduced ischemic tissue damage along with decreased leukocyte recruitment but increased numbers of M2-like polarized macrophages, thereby enhancing tissue regeneration, repair, and healing.

## 1. Introduction

Ischemic events in tissues and cells play a pivotal role in medicine, as cardiovascular diseases like myocardial infarction, peripheral artery disease, and strokes are the leading causes of mortality globally [1]. Ischemic tissue undergoes a sequence of events upon limited blood supply, triggering cellular distress marked by energy depletion, metabolic dysfunction, and eventually cell death. During the inflammatory response upon ischemia, the complement system is activated and immune cells migrate into the affected tissue. Achieving a delicate balance in the immune response becomes crucial to mitigate tissue damage [2].

The complement system is a crucial component of innate immunity. Consisting of a variety of proteolytic and regulatory proteins, chemoactive peptides, and membrane-bound receptors [3], it promotes the elimination of pathogens but also sensitizes autologous cells through opsonization, promotes the recruitment of immune cells, and directs lysis of target cells [4]. The complement cascade is initiated through three distinct activation pathways: the classical, the alternative, and the lectin pathway that all lead to the cleavage of C3 into C3b and C3a by the C3 convertases. Microbial cell-bound C3b plays a pivotal role as a potent marker for opsonization, enhancing the phagocytosis of pathogens and altered cells. Simultaneously, C3a and C5a, resulting from the cleavage of C5 into C5a and C5b, function as anaphylatoxins/chemoattractants, triggering subsequent immune cell responses [5]. In the final stage, complement activation triggers the assembly of the terminal membrane attack complex (MAC) through the sequential binding of the complement proteins C5b, C6, C7, C8, and C9 to form a membranolytic pore. Embedding itself into lipid bilayers, it initiates the formation of transmembrane poly-C9 channels which disrupt cellular integrity [6]. This possibility of cell lysis serves as the ultimate killing mechanism against pathogens [4].

Besides its contribution to physiological defense, the complement system is also involved in several pathophysiological processes including autoimmune diseases, neurodegenerative disorders, and ischemia-reperfusion injury [3,7,8]. In angiogenesis, the complement system modulates capillary growth, e.g., through the chemoattractant peptides C3a and C5a but also via the MAC [9,10]. Some studies have shown that dysregulated complement activation contributes to aberrant vessel growth in conditions of chronic inflammation like age-related macular degeneration or diabetic retinopathy [11,12]. However, the complement system has also been demonstrated to impair angiogenesis and neovascularization under ischemic conditions [13,14,15].

Cobra venom factor (CVF), derived from cobra venom, is a high molecular weight protein and functionally homologous to C3b, yet with a much higher stability. By binding factor B, which is then cleaved by factor D, the stable C3/C5 convertase CVF-Bb is formed, which drives the alternative pathway of the complement system [16]. This C3/C5 convertase possesses unique properties that allow it to further initiate, activate, and inhibit the complement system by C3 depletion [8]. Hence, CVF serves as a valuable experimental tool for understanding the alternative pathway and investigating complement activation and inhibition under various pathological conditions.

Angiogenesis, the process of generating new capillaries from existing blood vessels, crucially involves endothelial cell proliferation, migration, and differentiation. Under normal conditions, a delicate balance between pro- and anti-angiogenic factors maintains vascular stability. However, in response to tissue damage or injury, the release of potent pro-angiogenic factors such as vascular endothelial growth factor (VEGF), fibroblast growth factor (FGF), and angiopoietins may trigger angiogenesis. Endothelial cells respond by proliferating and migrating to the injury site, initiating sprouts that elongate into new vessels, fostering tissue repair and regeneration by delivering oxygen, nutrients, and immune cells [17,18]. In ischemic tissue damage, like in myocardial infarction or peripheral artery disease, the formation of new capillaries becomes vital to clear cellular debris and support tissue recovery. However, reinstating blood supply requires collateral artery growth (arteriogenesis) to compensate for occluded arteries [19,20].

VEGF-A emerges as a primary driver of angiogenesis [21,22]. Beyond endothelial cells, platelets and leukocytes, notably monocytes, significantly contribute to VEGF-A supply, fostering an angiogenic microenvironment. Macrophages are actively engaged in diverse aspects of vessel formation, contributing to angiogenesis through various mechanisms, including the release of pro-angiogenic factors and tissue-remodeling matrix metalloproteinases [23,24]. Leukocyte-mediated angiogenesis relies on their recruitment, adhesion, and migration across endothelial barriers, facilitated by molecules like selectins and notably ICAM-1, essential for orchestrating neo-vascularization [25,26].

The role of the complement system in angiogenesis and ischemic tissue damage is not fully understood. Thus, this study aims to investigate the influence of complement activation on angiogenesis and tissue repair by utilizing CVF as an experimental tool in vivo.

## 2. Materials and Methods

### 2.1. Animals and Experimental Procedures

All experimental procedures and animal care were conducted in accordance with German animal legislation guidelines and approved by the Bavarian Animal Care and Use Committee (ethical approval code: ROB-55.2Vet-2532.Vet_02-17-99). C57BL/6J mice from Charles River Laboratory (Sulzfeld, Germany) were purchased. Male mice aged 8–10 weeks underwent a surgical procedure under anesthesia (combination of fentanyl (0.05 mg/kg, CuraMED Pharma, Karlsruhe, Germany), midazolam (5.0 mg/kg, Ratiopharm GmbH, Ulm, Germany), and medetomidine (0.5 mg/kg, Pfister Pharma, Berlin, Germany)) in which the right femoral artery was ligated to induce ischemia and stimulate angiogenesis in the gastrocnemius muscle. The left leg served as a sham-operated control, following established protocols [27]. Despite influencing the coagulation cascade through the application of CVF, no relevant bleeding complications were observed either intraoperatively or in the further progress of the experiments. As previously described [15], to study vascular cell proliferation, bromodeoxyuridine (BrdU), a proliferation marker, was administered daily via intraperitoneal injection (1.25 mg, Sigma-Aldrich, St. Louis, MO, USA) dissolved in 100 µL phosphate-buffered saline (PBS, 148 mM Na^+^, 1.8 mM K^+^, pH 7.2), starting directly after femoral artery ligation. Then, 24 h before femoral artery ligation, 12.5 µg of Cobra venom factor (CVF, REF A600, Quidel Co., Athens, OH, USA) diluted in 50 µL PBS was injected into the mice intraperitoneally (i.p). According to the established protocols, tissue samples were collected at 7 days after femoral artery ligation, with a group size of *n* = 5 for each group [28]. After sacrifice, the mice were perfused with adenosine buffer (Sigma-Aldrich, Taufkirchen, Germany). Adenosine buffer was composed of 1% adenosine and 5% bovine serum albumin (BSA, Sigma-Aldrich, Taufkirchen, Germany) dissolved in PBS and subsequently fixed with 3% paraformaldehyde (PFA, Merck, Darmstadt, Germany) for cryopreservation. The gastrocnemius muscles were then removed, kept overnight in a 30% sucrose solution, stored in vinyl molds (REF 4566, Sakura Finetek, Torrance, CA, USA) in Tissue Tek^®^ (REF 4583, Sakura Finetek, Torrance, CA, USA), and preserved at minus 80 °C. To verify effective depletion of the complement system after CVF administration, serum complement activity was monitored 24 h after CVF administration using a CH_50_ assay. Therefore, 24 h after CVF administration, blood was collected and centrifuged at 4 °C for 10 min at 706× *g* and the supernatant was stored at −80 °C. Then, following established protocols, the CH_50_ assay was performed using the collected tissue [29]. The CH_50_ values shown are the reciprocal of the amount of serum capable of lysing 50% of sheep erythrocytes. Specimens without lytic activity were defined at 0 CH_50_ units/mL.

### 2.2. Histological and Immunofluorescence Analysis

Cryosections of the gastrocnemius muscle, 8 µm thick, were stained for immunohistological analysis. Tissue samples obtained 7 days after femoral artery ligation were used to stain for endothelial cells, leukocytes, and macrophages. To stain BrdU, the sections were treated with 1N HCl at 37 °C for 30 min to expose BrdU in the nuclei. Subsequently, they were blocked with 10% goat serum dissolved in 4% BSA PBS/0.1% Tween-20 for 1 h at room temperature (RT) and incubated overnight at 4 °C with anti-BrdU antibody (1:50, Abcam, Cambridge, UK, ab6326). The sections were then incubated with goat anti-rat Alexa Fluor^®^ 546 antibody (1:100, Thermo Fisher, Rockford, IL, USA, A11081) as the secondary antibody. To stain endothelial cells (CD31^+^/CD45^−^) and leukocytes (CD45^+^), the sections were further incubated with Alexa Fluor^®^ 647 anti-mouse CD31 antibody (1:50, BioLegend, San Diego, CA, USA, 102516) and anti-CD45-Alexa Fluor^®^ 488 antibody (1:100, Thermo Fisher, Rockford, IL, USA, 11-0451-85). Additionally, anti-CD45^−^Alexa Fluor^®^ 488 antibody (1:100, Thermo Fisher, 11-0451-85) was used to label leukocytes, while anti-CD68-Alexa Fluor^®^ 488 antibody (1:200, Abcam, ab201844) and anti-mannose receptor C type 1 (anti-MRC1) antibody (1:200, Abcam, ab64693) were used to label macrophages and evaluate macrophage polarization. The MRC1-antibody donkey anti-rabbit-Alexa Fluor^®^ 546 antibody (1:200, Thermo Fisher, A10040) served as a secondary antibody. All tissues were stained with DAPI (1:1000, Thermo Fisher, 62248) to label nucleic DNA. For mounting the stained slides, Dako mounting medium (Dako, Agilent, Santa Clara, CA, USA) was used. The mounted tissue from CVF-treated and control mice (*n* = 5 per group and timepoint) was analyzed by counting cells in an ischemic area of 1.5 mm^2^ (CD45^+^ cells, CD68^+^ cells). Muscle fibers were identified by their auto-fluorescent signal and the capillary-to-muscle fiber ratio was used as an angiogenesis index. The capillary-to-muscle fiber ratio was calculated as previously described [30]) and CD31^+^/CD45^−^/DAPI^+^ cells were counted as capillaries. TUNEL staining was performed with ApopTag^®^ Plus Fluorescein in Situ Apoptosis Detection Kit (EMD Millipore Corp., Burlington, MA, USA) following the manufacturer’s instructions. Images for cell counting were captured using a Leica DM6 B epifluorescence microscope with a 20x objective lens. For CD68/MRC1 as well as CD31/BrdU/CD45 staining and CD45 quantification, 5 pictures (630 × 475 µm) per muscle were analyzed. Cell counting analysis was performed using the open-source program ImageJ. For the hematoxylin and eosin (H&E) and TUNEL staining, 8 µm thick cryo-fixed gastrocnemius muscle sections (*n* = 5 per group) were used and the respective total necrotic and apoptotic area (%) of the tissue slices were quantified using ImageJ 1.54h.

## 3. Results

To investigate the role of the complement system in ischemia and angiogenesis, we induced tissue ischemia in the gastrocnemius muscles of CVF-treated compared to control mice by ligating the right femoral artery (FAL) distal of the deep femoral branch. The left sham-operated legs served as internal negative controls. Based on empirical values, gastrocnemius muscles were collected for (immuno-) histological analyses 7 days after FAL [15,27,31,32]. The depletion of the complement system by the application of CVF was confirmed using a CH_50_ assay (Supplementary Materials, Figure S6).

### 3.1. CVF Treatment Leads to Reduced Ischemic Tissue Damage

**Figure 1:**
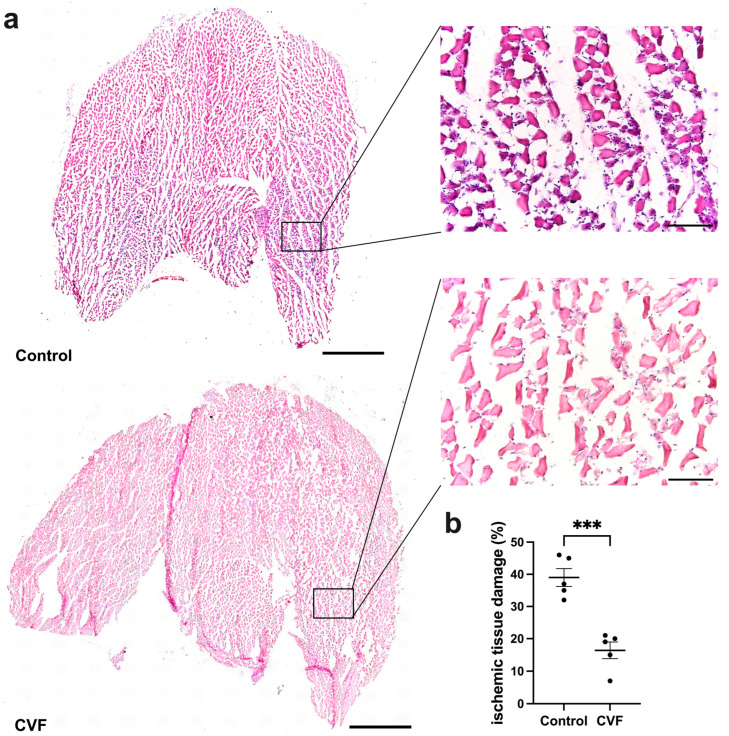
Tissue damage is reduced in CVF-treated mice. (**a**) Representative H&E-stained images of gastrocnemius muscles (left) with magnifications (right) of the areas shown in the black boxes of control mice (upper images) and cobra venom factor-treated mice (lower images) taken 7 days aFAL. (**b**) Scatter plot presenting the area of ischemic tissue damage (%) in control and CVF-treated mice 7 days after femoral artery ligation (aFAL). One complete sectional area was assessed per mouse per group. The data represent means ± SEM, with *n* = 5 per group. *** *p* ≤ 0.001 (control vs. CVF) determined by unpaired Student’s *t*-tests. The scale bars represent 1000 µm (overview) and 100 µm (detail).

**Figure 2:**
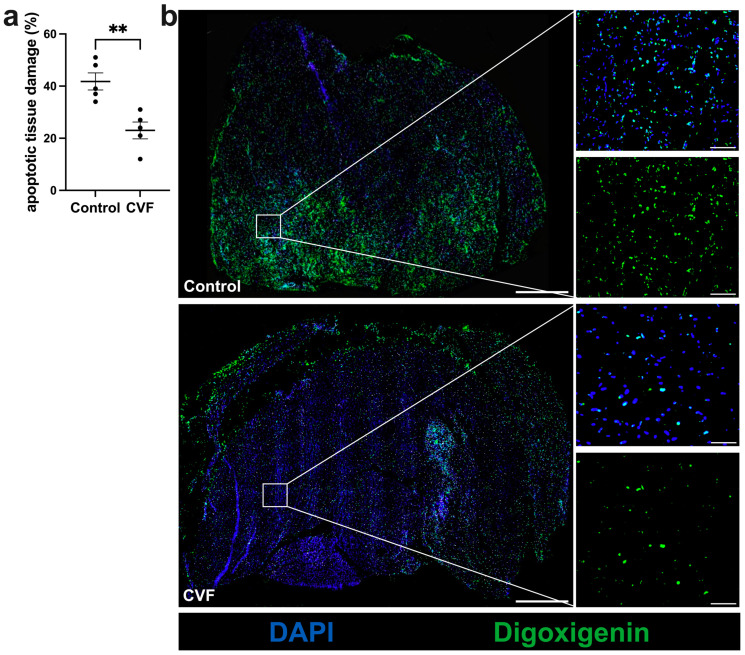
TUNEL staining revealed decreased apoptosis in CVF-treated mice compared to control mice. (**a**) The scatter plot depicts the apoptotic area (%) in control and CVF-treated mice 7 days after femoral artery ligation (aFAL). Analysis was conducted on one complete sectional area per mouse per group. The data presented are means ± SEM, with *n* = 5 per group. ** *p* ≤ 0.01 (control vs. CVF) determined by unpaired Student’s *t*-tests. (**b**) Magnified images (right) of the areas shown in the white boxes of the representative images (left) of TUNEL-stained gastrocnemius muscles from control mice (upper images) and CVF-treated mice (lower images) showing apoptotic cells (green) at 7 days aFAL. Scale bars: 1000 µm (overview), 100 µm (detail).

H&E staining and TUNEL assay of tissue specimens confirmed ischemic damage in the gastrocnemius muscles of both CVF-treated and control mice. However, CVF-treated mice exhibited significantly decreased ischemic and apoptotic damage compared to control mice (Figure 1a,b and Figure 2a,b). The gastrocnemius muscles from sham-operated legs showed no signs of ischemic damage (Supplementary Materials, Figures S1 and S2).

### 3.2. CVF-Treated Mice Show a Reduced Capillarity

Next, we examined whether complement activation/depletion by administration of CVF affects the process of angiogenesis in ischemic muscle tissue. To this end, a quadruple immunofluorescence staining (CD31/CD45/BrdU/DAPI) was performed on gastrocnemius muscles isolated 7 days after the FAL procedure. CD31 antibody was used as a marker for endothelial cells, CD45 antibody labels leukocytes, and BrdU stains proliferating cells. As CD31 is not exclusively expressed on endothelial cells but might be expressed on leukocytes as well, CD31^+^/CD45^+^ cells were excluded from endothelial counts.

**Figure 3:**
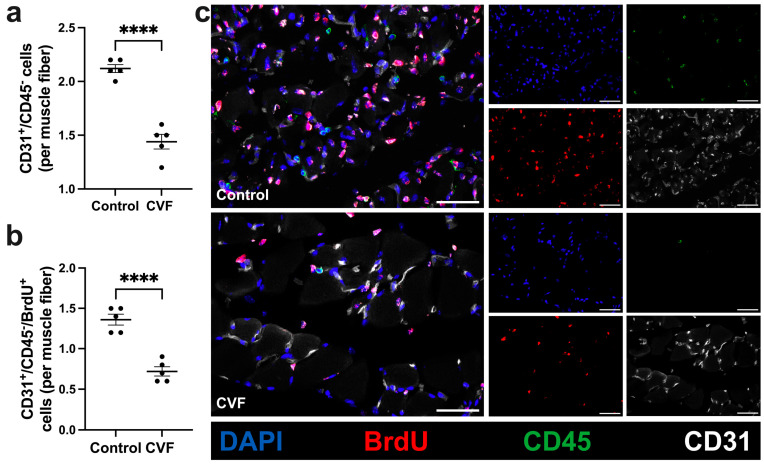
CVF treatment led to reduced capillarity. Scatter plots illustrate the number of (**a**) endothelial cells (CD31^+^/CD45^−^) and (**b**) proliferating endothelial cells (CD31^+^/CD45^−^/BrdU^+^) per muscle fiber of ischemic gastrocnemius muscles in control and CVF-treated mice 7 days after femoral artery ligation (aFAL). The data presented are means ± SEM, with *n* = 5 per group. A defined ischemic area (1.5 mm^2^) of muscle tissue was analyzed per mouse. **** *p* ≤ 0.0001 (control vs. CVF) determined by unpaired Student’s *t*-tests. (**c**) Representative immunofluorescence images of analyzed ischemic gastrocnemius muscles from control (upper images) and CVF-treated mice (lower images) at 7 days aFAL. Single channel pictures (small images on the right) and merged pictures (large images on the left) show endothelial cells (CD31 in gray), proliferating cells (BrdU in red), leukocytes (CD45 in green), and the nuclei (DAPI in blue). Scale bars: 50 µm.

CVF-treated mice showed a significantly decreased capillarity compared to control mice (Figure 3a,c) as seen in the number of CD31^+^/CD45^−^ cells. Furthermore, treatment with CVF led to a reduced number of proliferating endothelial cells (CD31^+^/CD45^−^/BrdU^+^) per muscle fiber compared to control mice (Figure 3b,c). Non-ischemic gastrocnemius muscles from sham-operated legs of CVF-treated and control mice did not differ in the capillary-to-muscle fiber ratio (Supplementary Materials, Figure S3).

### 3.3. Treatment with CVF Leads to Reduced Leukocyte Infiltration

**Figure 4:**
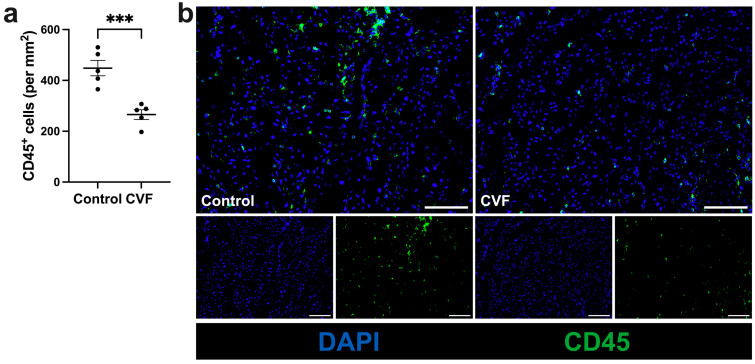
CVF treatment reduced leukocyte infiltration in ischemic gastrocnemius muscles. (**a**) The scatter plot illustrates the number of leukocytes (CD45^+^) per square millimeter (mm^2^) in ischemic gastrocnemius muscles isolated 7 days after femoral artery ligation (aFAL). The data presented are means ± SEM, with *n* = 5 per group. Analysis was conducted on a defined ischemic area (1.5 mm^2^) of muscle tissue per mouse. *** *p* ≤ 0.001 (control vs. CVF) determined by unpaired Student’s *t*-tests. (**b**) Representative immunofluorescence images of analyzed gastrocnemius muscles from control (left image) and CVF-treated mice (right image) at 7 days aFAL. Leukocytes in single-channel pictures (lower images) and merged pictures (upper images) were labeled with CD45 antibodies (green) and the nuclei were labeled with DAPI (blue). Scale bars: 100 µm.

Further studies revealed that the number of leukocytes (CD45^+^) that infiltrated the muscle tissue on day 7 after FAL was significantly lower in the ischemic tissues of CVF-treated mice compared to control mice (Figure 4a,b). The examinations of the sham-operated gastrocnemius muscles showed a low leukocyte count without any variation in quantity (Supplementary Materials, Figure S4).

### 3.4. CVF-Treated Mice Show a Lower Number of Macrophages with a Higher Percentage of M2-like Polarization

**Figure 5:**
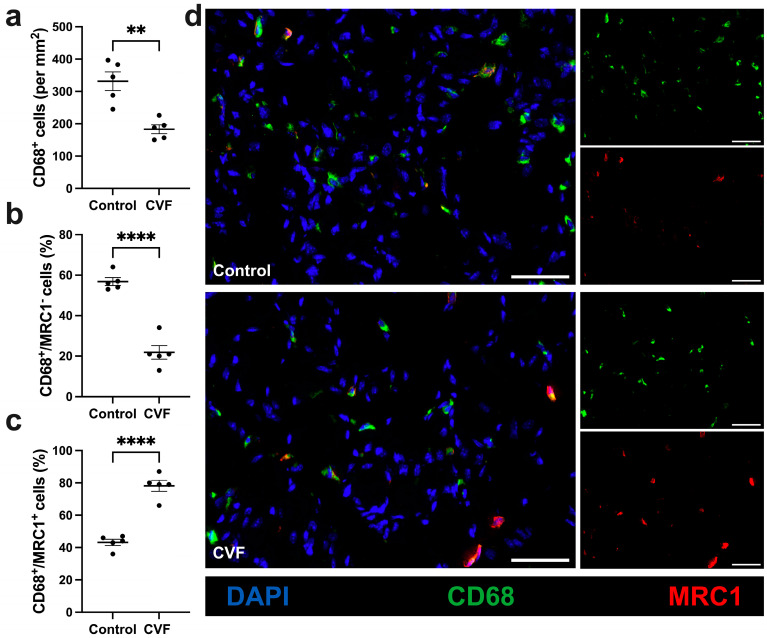
CVF treatment promotes M2-like polarization in ischemic muscle tissue. The scatter plot displays (**a**) the total number of macrophages (CD68^+^) per square millimeter (mm^2^) and the percentage of (**b**) M1-like polarized macrophages (CD68^+^/MRC1^−^) and (**c**) M2-like polarized macrophages (CD68^+^/MRC1^+^) in ischemic gastrocnemius muscle tissue at 7 days after femoral artery ligation (aFAL). Data are shown as means ± SEM, with *n* = 5 per group. ** *p* ≤ 0.01 and **** *p* ≤ 0.0001 (control vs. CVF) determined by unpaired Student’s *t*-tests. (**d**) Representative immunofluorescence images of analyzed ischemic gastrocnemius muscles from control (upper image) and CVF-treated mice (lower image) at 7 days aFAL. In single-channel pictures (small images on the right) and merged pictures (large images on the left), macrophages were labeled with CD68 antibody (green) and M2-like polarized macrophages were labeled with MRC1 antibody (red). Nuclei were labeled in merged images with DAPI (blue). Scale bars: 50 µm.

Focusing on macrophages and their polarization, we used CD68 as a marker for macrophages and MRC1 for M2-like polarized macrophages. In the tissue sections of interest, a reduced total number of macrophages per mm^2^ was found but an increased percentage of M2-like polarized macrophages (CD68^+^/MRC1^+^ cells) was seen in tissue sections of CVF-treated mice compared to control mice (Figure 5a,c,d). The control mice, however, showed a higher amount of CD68^+^/MRC1^−^ cells (Figure 5b,d). In the non-ischemic muscles of sham-operated mice, no difference was found between CVF-treated mice and control mice either regarding the number of macrophages or their polarization (Supplementary Materials, Figure S5).

## 4. Discussion

In this study, the influence of cobra venom factor (CVF) as a complement-depleting tool was studied on angiogenesis and ischemic muscle tissue damage using a hind limb model of ischemia. Our results demonstrate that CVF-induced complement activation is associated with decreased ischemic tissue damage, along with a reduced recruitment and infiltration of CD45^+^ and CD68^+^ cells. Moreover, CVF-treated mice exhibited more macrophages polarized towards the pro-angiogenic regenerative M2-like phenotype (CD68^+^/MRC1^+^) compared to control mice. The results of this study indicated that CVF as a complement activator (or complement depletion, respectively) plays a key role in the reduction in ischemic muscle tissue damage by facilitating the switch of macrophages towards the anti-inflammatory and regenerative M2-like polarization.

**Figure 6:**
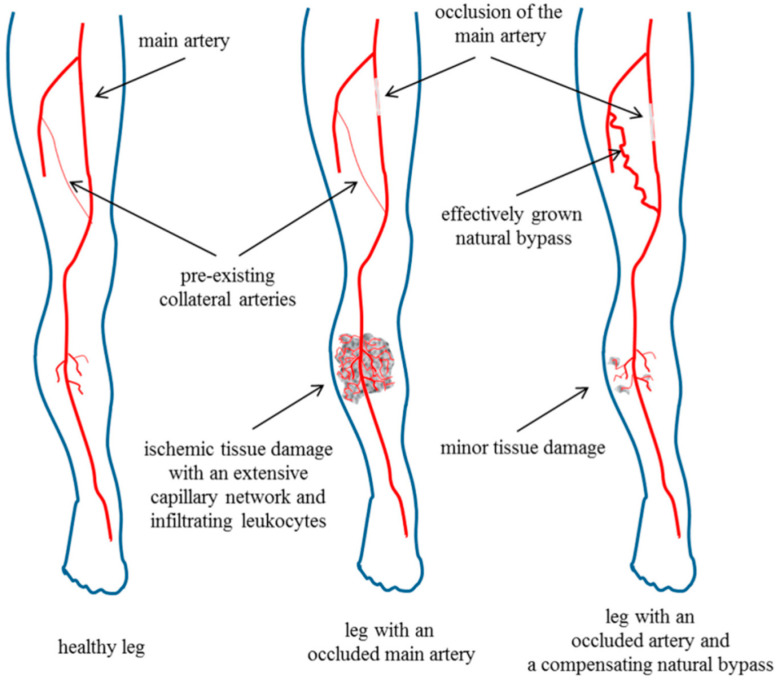
The connection between arteriogenesis and angiogenesis. The left picture shows a healthy leg with pre-existing collateral arteries. The leg in the middle displays an occluded main artery with ischemic tissue damage and a capillary network in the lower leg as a result of the occlusion in the upper leg. The leg on the right side shows the effect of arteriogenesis to decrease the extent of ischemia in the lower leg (Image from Chillo et al. [33]).

The occlusion of the femoral artery leads to increased shear stress in collateral arteries in the upper leg, eventually resulting in arteriogenesis (distal to the occlusion) as a compensatory mechanism to decrease ischemic tissue damage in the lower leg [33]. As a consequence, the blood flow is redirected through collateral vessels to bypass the blockage and supply of oxygen and nutrients to distal tissues. As reported by Chillo et al. (2016), an improvement in arteriogenesis leads to a decrease in ischemic tissue damage in the gastrocnemius muscle [33]. This connection between arteriogenesis and angiogenesis in the murine hind limb model, which needs to be considered in any investigation, is shown in Figure 6 (adapted from Chillo et al. (2016) [33]). Previous studies by Götz et al. (2022) demonstrated that treatment with CVF 24 h prior to femoral artery ligation significantly enhances the perfusion recovery 7 days aFAL. Furthermore, an increased number of proliferating vascular cells and an increased luminal diameter of the grown collateral vessels could be observed, indicating that CVF (complement depletion) has a positive impact on arteriogenesis [29]. Based on H&E staining and the TUNEL assay, we found a reduction in the degree and area of ischemic tissue damage as evidenced by reduced apoptosis and cell debris accumulation in CVF-treated mice.

The role of the complement system in angiogenesis has been investigated in several studies [34]. In order to quantify the activity of the complement system in the context of an inflammatory response to an ischemic stimulus, the serum concentration of complement factor C3b can be assessed. In an experimental model of mouse renal ischemia/reperfusion injury, a significant increase in plasma C3b concentration was observed 24 h after reperfusion, which returned to the values of the sham control group 7 days after reperfusion [35]. The influence of arteriogenesis in the upper leg on ischemic damage is a significant difference to previous studies on the influence of CVF on ischemic damage. As previously described, various studies demonstrated the role of the complement system in the inflammatory processes after reperfusion and the protective effect of CVF on ischemia-reperfusion injuries [2,36,37,38,39]. However, while in models of ischemia-reperfusion injury blood flow is restored after a defined time interval by reopening at hoc the previously occluded artery, reperfusion after FAL only occurs over time due to the growth of natural collaterals. Importantly, the latter is not associated with reperfusion injury as is the case when occluded arteries are reopened acutely.

By depleting complement components, CVF can limit downstream inflammatory responses and the formation of the membrane attack complex (MAC) thus attenuating complement-dependent tissue damage and inflammation. The reduced damage shown in this study is thus in line with the previous findings of Chillo et al. (2016) and Götz et al. (2022). Moreover, an increased percentage of anti-inflammatory and regenerative M2-like macrophages were detected in ischemic gastrocnemius muscle 7 days aFAL. The regenerative M2-like macrophages can resolve inflammatory conditions, provide tissue repair, and facilitate tissue remodeling [40,41]. Hence, they constitute another positive factor for the reduced tissue damage shown in this study.

Furthermore, we investigated the infiltration of leukocytes, which we found to be reduced in CVF-treated mice. Complement components C3a and C5a bind to their respective receptors C3aR and C5aR on the surface of leukocytes. They act as chemoattractants for leukocytes and stimulate leukocyte migration/recruitment to the site of injury by inducing chemotaxis [4,9,42]. Complement activation also enhances the adhesion of leukocytes to endothelial cells by upregulating the expression of adhesion molecules, such as intercellular adhesion molecule-1 (ICAM-1) and vascular cell adhesion molecule-1 (VCAM-1), on endothelial cells [43,44]. Together, this promotes the initial attachment of leukocytes to the endothelium, allowing them to undergo rolling interactions coupled with subsequent firm adhesion necessary for extravasation into the inflamed tissue. With respect to CVF treatment, the overall reduced leukocyte recruitment in our study may be potentially due to the interplay between inhibition of complement activation and downstream regulatory mechanisms. Depleting the complement by CVF might impede the generation of these chemotactic factors and attenuate the expression of adhesion molecules on endothelial cells leading to reduced leukocyte recruitment, rolling, and infiltration into the tissues. This indicates that by dampening the overall immune response, CVF attenuates excessive inflammation and prevents further tissue damage.

Next, we focused on the influence of CVF on macrophage infiltration and polarization. Our results revealed that CVF treatment led to a decrease in macrophage infiltration; however, with a higher number of anti-inflammatory and pro-regenerative M2-like polarized macrophages compared to the control group. This was evidenced by MRC1, a marker associated with the M2 phenotype.

The presence of macrophages and their polarization play a crucial role in the context of angiogenesis in ischemic tissue [23]. Depending on the stimulus, macrophages differentiate into either the pro-inflammatory (M1-like, CD68^+^/MRC1^−^) or the anti-inflammatory and regenerative (M2-like, CD68^+^/MRC1^+^) phenotype [45]. Both macrophage phenotypes are crucial for sufficient angiogenesis to occur. Initially, M1-like macrophages provide pro-angiogenic factors, such as VEGF and TNF-α, at an early stage of angiogenesis, while M2-like macrophages are decisive in the further process of tissue healing, regeneration, and remodeling [40,41,46,47,48].

A study by Götz et al. (2021) in which angiogenesis was investigated in C3-deficient mice, showed that the absence of the complement system leads to an increased percentage of M2-like polarized macrophages [15]. In addition, another study of our research group indicated that treatment with CVF leads to a significantly increased number of perivascular M2-like polarized macrophages in the adductor muscle of mice during the process of arteriogenesis [29]. Since we also observed this effect in the present setting, the depletion of the complement system by CVF and the subsequent consumption of C3 decreases the anaphylatoxins C3a and C5a in the circulation and thus might facilitate a shift towards the M2 macrophage phenotype in a similar manner. Studies have also shown that C3a modulates inflammation by inducing macrophage polarization to a pro-inflammatory phenotype [49,50]. Since the activation of the complement system by CVF leads to a consumption of complement (C3) and therefore depletion of complement activity, the increased M2-like polarization of macrophages in this study is consistent with the literature mentioned above.

Moreover, treatment of mice with CVF in our study resulted in a decreased capillarity compared with the control group, evidenced by a lower number of CD31 positive cells per muscle fiber. The number of proliferating endothelial cells (CD31^+^/BrdU^+^ cells) was also significantly reduced in CVF-treated mice compared to the control group. A study by Goetz et al. (2021) showed that the absence of complement factor C3 in knockout mice (C3 -/- mice) leads to improved angiogenesis [15]. Since the treatment of mice with CVF leads to a depletion of the complement system, these results appear to be in contrast with recent findings. However, the initial activation of the complement system by CVF represents a different physiological condition compared to C3 knockout.

Furthermore, the correlation of arteriogenesis and angiogenesis might play a decisive role in this context: treatment with CVF leads to improved arteriogenesis (as shown in the study of Götz et al.), resulting in a less inflammatory environment and—as shown in this study—lower ischemic tissue damage. Chillo et al. (2016) demonstrated that reduced tissue damage is correlated with a lower ischemic stimulus for angiogenesis [33]. Hence, the pro-angiogenic stimulus in CVF-treated mice on endothelial cells may be attenuated.

With regard to a therapeutic benefit of CVF for the treatment of cardiovascular occlusive diseases, a detailed understanding of the metabolic, pharmacodynamic, and toxicological properties of CVF is of great clinical relevance. As described above, CVF is a complement activating protein that was discovered in the venom of the Indian cobra *Naja naja* and has the property of complement activation due to its high structural similarity to the complement component C3 [51,52,53]. After application of CVF into the organism, the enzyme CVF, Bb, which is resistant to inactivation by factor H and I, is produced, which is the basis for depletion of the complement system [16]. With the exception of cobras, CVF leads to depletion of the complement system in all vertebrates tested [54]. After it was shown that CVF can be safely administered for temporary complement depletion in laboratory animals, it became a widely used experimental tool [55]. The only relevant side effect in animal models caused by the strong complement activation after CVF administration was an acute inflammatory C5a-mediated damage of the lungs [56,57]. However, the damage to lung tissue caused by neutrophil activation and sequestration in the lungs was only temporary. After a humanized form of CVF was developed, therapeutic benefits were demonstrated in several areas, including myocardial ischemia reperfusion injury, age-related macular degeneration, and arthritis [37,58,59,60]. In addition, no C5-cleaving activity was observed with the use of humanized CVF, making its therapeutic use well tolerated and safe [61].

Taken together, the molecular mechanisms underlying the actions of CVF in ischemia and angiogenesis involve complex interactions with macrophages, leukocytes, and endothelial cells. Treatment with CVF to deplete the complement system in vivo leads to reduced ischemic tissue damage in the gastrocnemius muscle and a reduction in the recruitment of macrophages and leukocytes. Furthermore, it stimulates the switch of macrophages towards the anti-inflammatory M2-like polarization, facilitating the process of healing and regeneration.

## 5. Conclusions

The present study provides evidence that CVF treatment not only boosts arteriogenesis as shown in our previous report [29] but also has the potential to reduce apoptosis and tissue damage in the ischemic tissue. This effect is not only due to tissue salvage by means of arteriogenesis but is also likely to be achieved through regulation of inflammatory (complement-derived) mediators and the modulation of the immune response. The findings of this study suggest a critical role for cobra venom factor in ameliorating ischemic tissue damage.

## 6. Statistical Analyses

GraphPad Prism 8 (GraphPad Software, LA Jolla, CA, USA) was used for the analyses of all our data. Results are expressed as mean values ± standard error of the mean (SEM) and all values of *p* < 0.05 were considered statistically significant.

## Data Availability

The data presented in this study are available on request from the corresponding authors.

## Supplementary Materials

The following supporting information can be downloaded at https://www.mdpi.com/article/10.3390/biomedicines12020309/s1. Figure S1: Representative pictures of hematoxylin and eosin (H&E) stained sham-operated gastrocnemius muscles (left) with magnifications (right) of control (top) and Cobra venom factor (CVF) treated-mice (bottom) do not show ischemic tissue damage 7 days after sham operation; Figure S2: Scatter plot displays no apoptotic tissue damage of sham-operated control and CVF-treated mice 7 days after surgery; Figure S3: Sham operated gastrocnemius muscles of control and CVF-treated mice show no difference in capillarity and no proliferation of endothelial cells 7 days after sham operation; Figure S4: Representative pictures of control and CVF-treated mice display a low leukocyte count without any variation in quantity; Figure S5: Sham operated control and CVF-treated mice show low numbers of macrophages 7 days after surgery; Figure S6: A single dose of 12.5 µg of the cobra venom factor (CVF (1x)) causes the loss of serum hemolytic complement activity 24 hours after injection.
