# Supplementary material for: Treatment with Cobra Venom Factor Decreases Ischemic Tissue Damage in Mice
Source: Biomedicines. 2024 Jan 29;12(2):309. doi: 10.3390/biomedicines12020309 (PMC10886846; doi:10.3390/biomedicines12020309)
Supplement: Supplementary file 1 [file biomedicines-12-00309-s001.zip › biomedicines-2824693-supplementary.pdf]

biomedicines

Article

Treatment with Cobra Venom Factor Decreases Ischemic Tissue Damage in Mice

Sharon O. Azubuike-Osu[1,2,3,x], Amelie Kuhs[1,2,x], Philipp Götz[1,2], Anna Faro[1,2], Klaus T. Preissner[4], Christoph Arnholdt[1,2,†] and Elisabeth Deindl[1,2,*,†]

1 Walter-Brendel-Centre of Experimental Medicine, University Hospital, Ludwig-Maximilians-Universität München, 81377 Munich, Germany; sharon.azubuike-osu@med.uni-muenchen.de (S.O.A.-O.); amelie.kuhs@campus.lmu.de (A.K.); p.goetz@med.uni-muenchen.de (P.G.); anna.braumandl@web.de (A.F.); christoph.arnholdt@med.uni-muenchen.de (C.A.); Elisabeth.Deindl@med.uni-muenchen.de (E.D.)
2 Biomedical Center, Institute of Cardiovascular Physiology and Pathophysiology, Ludwig-Maximilians-Universität München, 82152 Planegg-Martinsried, Germany
3 Department of Physiology, Faculty of Basic Medical Sciences, College of Medicine, Alex Ekwueme Federal University Ndufu Alike, Abakaliki 482131, Ebonyi, Nigeria; sharon.eboagwu@funai.edu.ng
4 Department of Cardiology, Kerckhoff-Heart Research Institute, Faculty of Medicine, Justus Liebig University, 35392 Giessen, Germany; klaus.t.preissner@biochemie.med.uni-giessen.de
* Correspondence: Elisabeth.Deindl@med.uni-muenchen.de; +49-(0)-89-2180-76504
x These authors contributed equally to this work
† These authors contributed equally to this work

Citation: Azubuike-Osu, S.O.; Kuhs, A.; Götz, P.; Faro, A.; Preissner, K.T.; Arnholdt, C.; Deindl, E. Treatment with Cobra Venom Factor Decreases Ischemic Tissue Damage in Mice. *Biomedicines* **2024**, *12*, 309. https://doi.org/10.3390/biomedicines12020309

Academic Editors: Bruno M. Fonseca and Shaker A. Mousa

Received: 29 December 2023
Revised: 23 January 2024
Accepted: 27 January 2024
Published: 29 January 2024

Copyright: © 2024 by the authors. Licensee MDPI, Basel, Switzerland. This article is an open access article distributed under the terms and conditions of the Creative Commons Attribution (CC BY) license

(https://creativecommons.org/licenses/by/4.0/).

Supplementary Materials

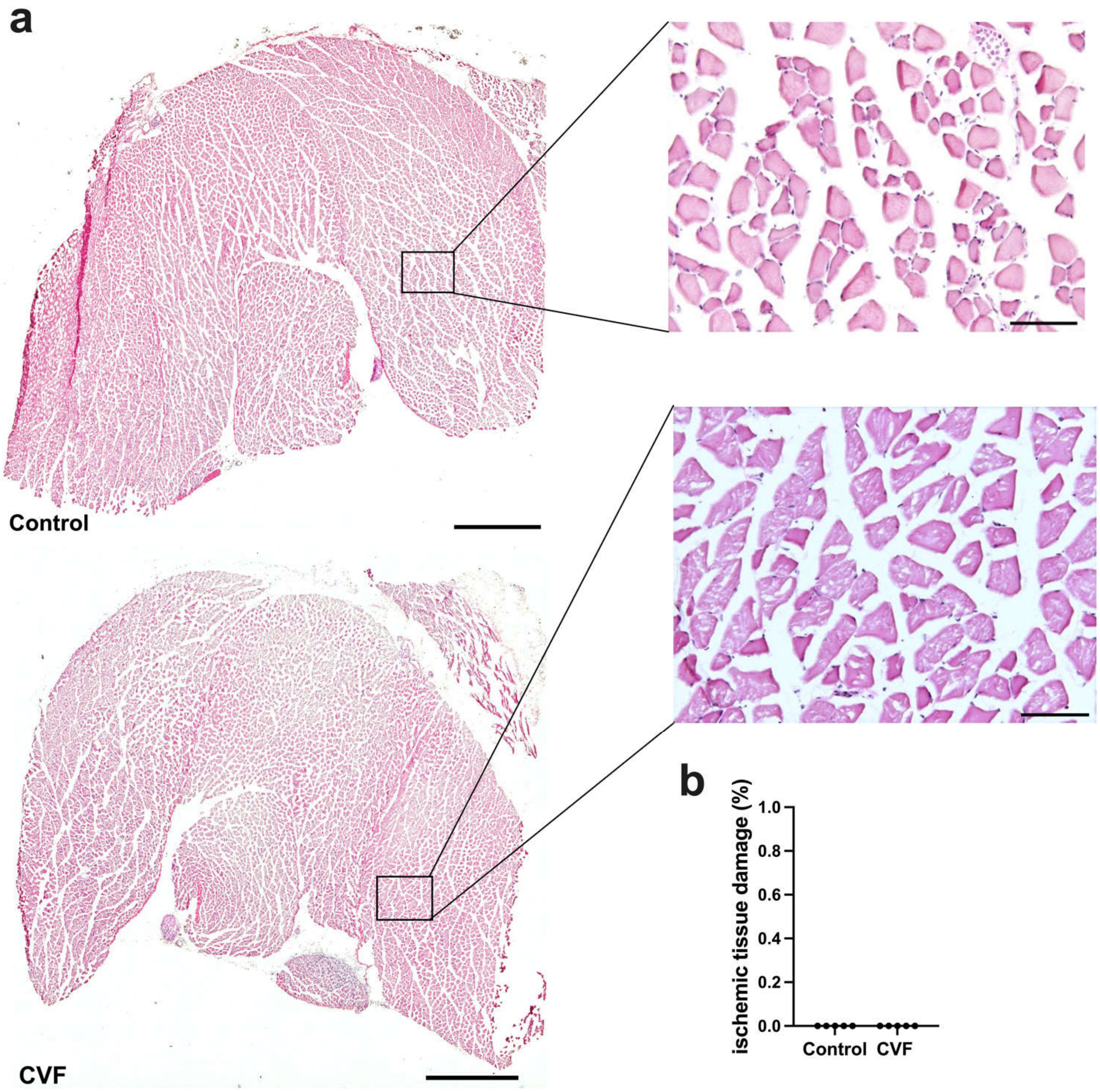

Figure S1. (**a**) Representative pictures of hematoxylin and eosin (H&E) stained sham-operated gastrocnemius muscles (left) with magnifications (right) of control (top) and Cobra venom factor (CVF) treated-mice (bottom) do not show ischemic tissue damage 7 days after sham operation. (**b**) Scatter plot presenting the area of ischemic tissue damage (%) of control and CVF treated mice 7 days after sham operation. One complete sectional area was analyzed per mouse per group. The data represent means ± SEM, with n = 5 per group (control vs. CVF) determined by unpaired Student's t-test. Scale bars: 1000 µm (overview), 100 µm (detail).

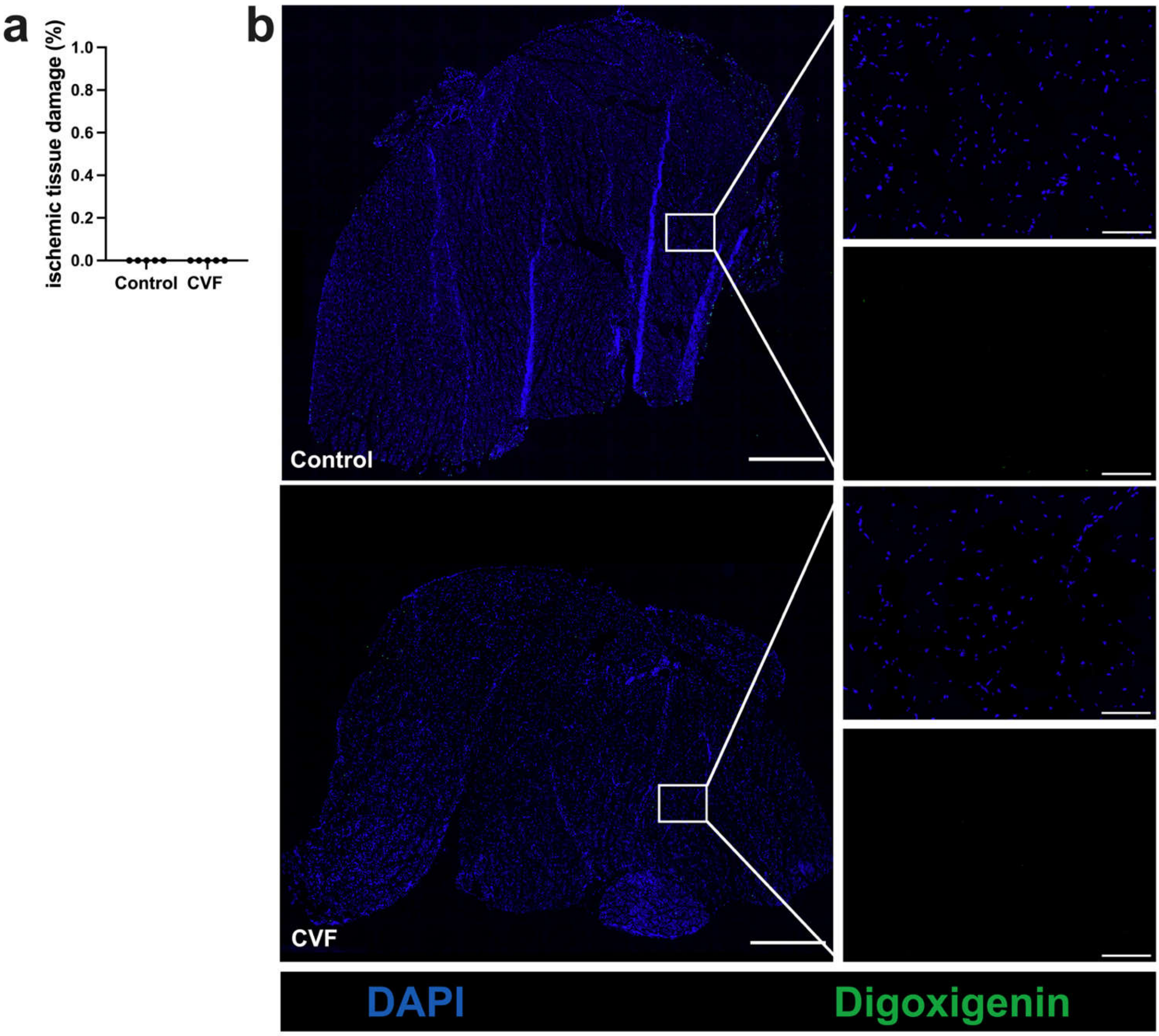

Figure S2. (**a**) Scatter plot displays no apoptotic tissue damage of sham-operated control and CVF-treated mice 7 days after surgery. Analyzed was one complete cross-section of the gastrocnemius muscle per mouse per group. The data shown are means ± SEM, with n = 5 per group (control vs. CVF) determined by unpaired Student's t-test. (**b**) Representative images (left) of Tunel stained gastrocnemius muscle from control (upper image) and CVF-treated mice (lower images) are shown in magnified images (right). Cells were labeled with antibodies against Digoxigenin (apoptotic cells, green) and DAPI (nuclei, blue). Scale bars: 1000 µm (overview), 100 µm (detail).

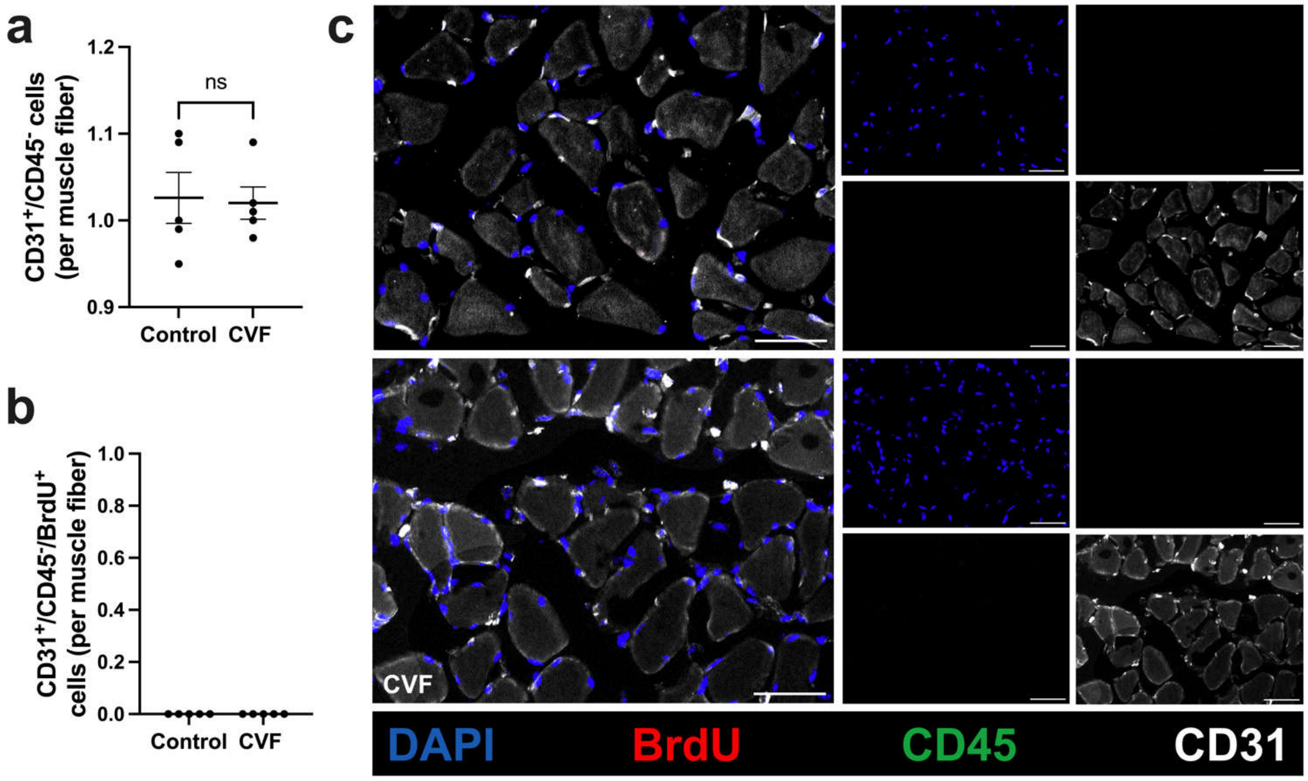

Figure S3. Sham operated gastrocnemius muscles of control and CVF-treated mice show no difference in capillarity and no proliferation of endothelial cells 7 days after sham operation. Scatter plots display the number of (**a**) $CD31^+/CD45^-$ endothelial cells and (**b**) proliferation endothelial cells ($CD31^+/CD45^-/BrdU^+$). The data presented are means ± SEM, with n = 5 per group. A defined area (1.5 mm^2) of muscle tissue was analyzed per mouse. ns $p > 0.05$ (control vs. CVF) determined by unpaired Student's t-test. (**c**) Representative images of control (top) and CVF treated mice (bottom). Cells were labeled with antibodies targeting BrdU (red, proliferation marker), CD45 (green, leukocytes), CD31 (white, endothelial cells) and DAPI (blue, nuclei). Scale bars: 50 µm.

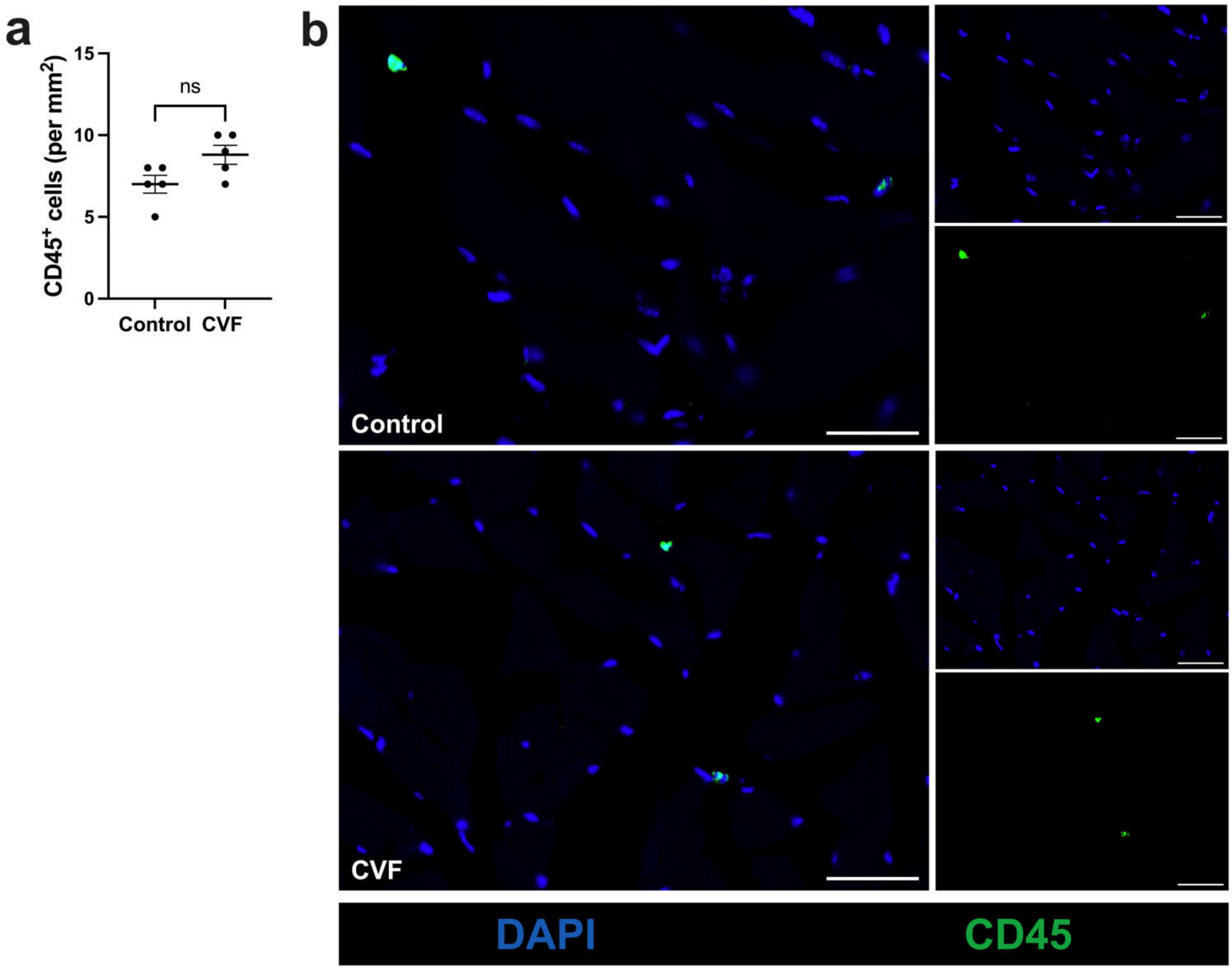

Figure S4. Representative pictures of control and CVF-treated mice display a low leukocyte count without any variation in quantity. (**a**) Scatter plot shows no significant difference in the number of leukocytes ($CD45^+$) per square millimeter between sham operated control and CVF-treated mice. The data presented are means ± SEM, with n = 5 per group. Analysis was conducted on a defined area (1.5 mm^2) of muscle tissue per mouse. ns $p > 0.05$ (control vs. CVF) determined by unpaired Student's t-test. (**b**) Representative immunofluorescence images of sham-operated legs of control (upper image) and CVF treated mice (lower image) 7 days after surgery. Antibodies directed against CD45 (leukocytes, green) and DAPI (nuclei, blue) were used to mark the cells. Scale bars: 50 µm.

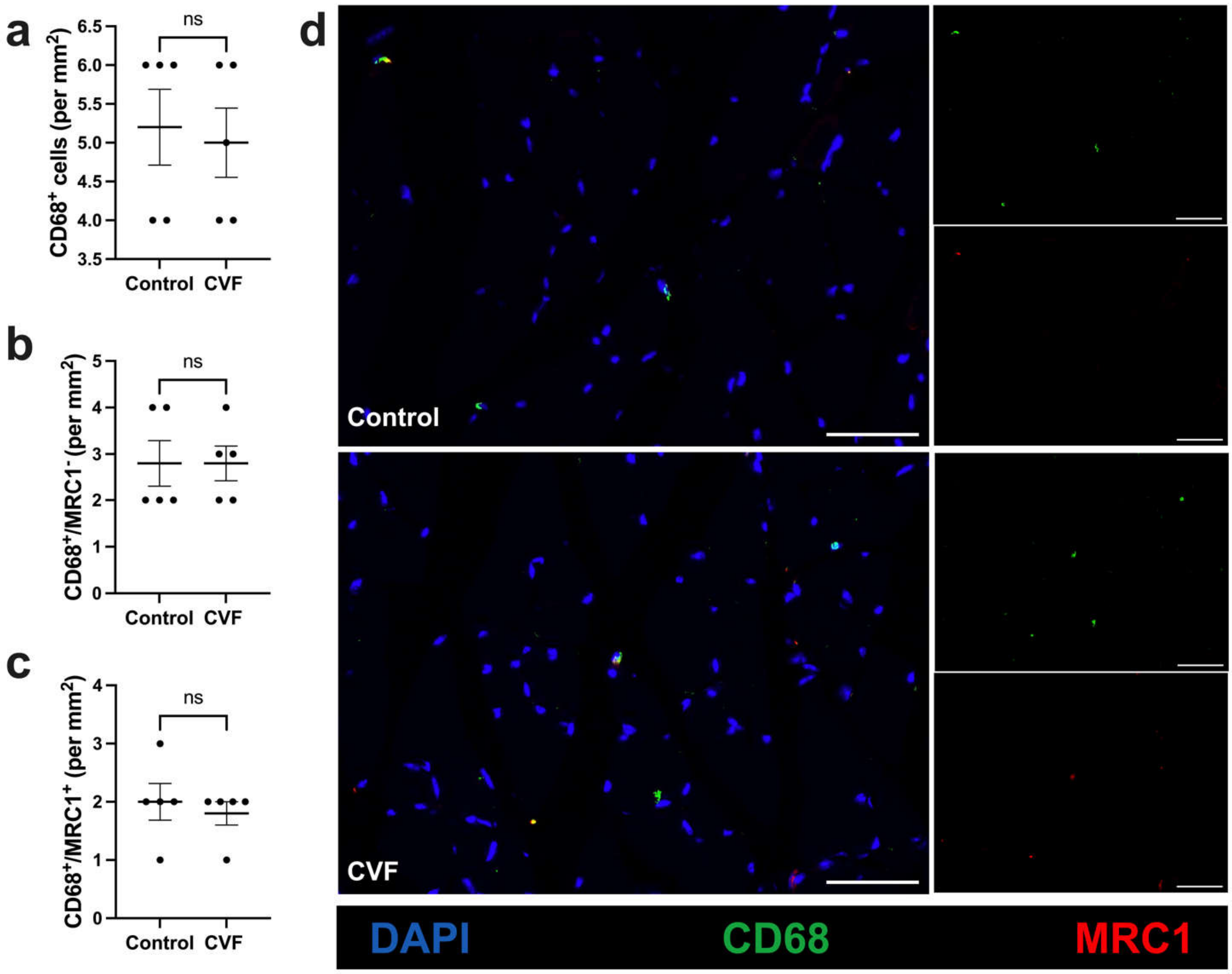

Figure S5. Sham operated control and CVF-treated mice show low numbers of macrophages 7 days after surgery. Scatter plots demonstrate (**a**) the number of macrophages ($CD68^+$), (**b**) the number of M1-like polarized macrophages ($CD68^+/MRC1^-$) and (**c**) the number of M2-like polarized macrophages ($CD68^+/MRC1^+$) per square millimeter (mm^2). Data are shown as means ± SEM, with n = 5 per group. ns p >0,05 (control vs. CVF) determined by unpaired Student's t-test. (**d**) Representative images of control (top) and CVF treated mice (bottom). Cells were labeled with antibodies targeting CD68 (green, macrophages), MRC1 (red, M2-like polarized macrophages) and DAPI (blue, nuclei). Scale bars: 50 µm.

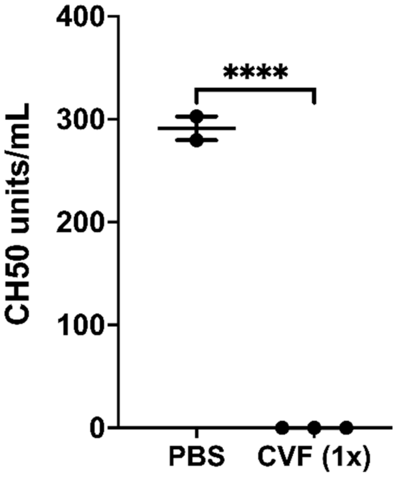

Figure S6. A single dose of 12,5µg of the cobra venom factor (CVF (1x)) causes the loss of serum hemolytic complement activity 24 hours after injection. Scatter plot displays the results of a CH50 assay. Data are shown as means ± SEM, with n = 2 for the PBS-treated control group and n = 3 for the CVF-treated group. **** $p < 0.0001$ (PBS vs. CVF (1x)) determined by unpaired Student's t-test. Adapted from Götz et al., 2022 [1], with permission of the authors.

REFERENCE

1. Götz, P., et al., *Cobra Venom Factor Boosts Arteriogenesis in Mice.* Int J Mol Sci, 2022. **23**(15).

Disclaimer/Publisher's Note: The statements, opinions and data contained in all publications are solely those of the individual author(s) and contributor(s) and not of MDPI and/or the editor(s). MDPI and/or the editor(s) disclaim responsibility for any injury to people or property resulting from any ideas, methods, instructions or products referred to in the content.
